# Supplementary material for: Content-rich biological network constructed by mining PubMed abstracts
Source: BMC Bioinformatics. 2004 Oct 8;5:147. doi: 10.1186/1471-2105-5-147 (PMC528731; doi:10.1186/1471-2105-5-147)
Supplement: Additional File 2 — The original results of the above study (non-essential files are deleted to keep the file size under the limit set by BMC bioinformatics). [file 1471-2105-5-147-S2.bz2 › chilibotAdditionalFile2/dip05/594ID8443157E10968/html/PRKACA_PKIA.html]

 


 **PRKACA** and **PKIA** 
  
Found 10 abstracts in PubMed, retrieved 05.  
 

 What does Google say? 
 PDF only 
| .edu only 

---

**Interactive relationship** (e.g. stimulation, inhibition, etc)

**Non-interactive relationship** (e.g. studied together, co-existance, homology, etc.)

- In contrast, Go6976 1 microM, a selective PKC alpha  [ **PRKACA** ]  and beta1 inhibitor, as well as the cAMP dependent protein kinase inhibitor  [ **PKIA** ] , H89 1 microM were without effect.  Ref: 10482351 Neurochem Int, 1999
